# Relations between Cardiac and Visual Phenotypes in Diabetes: A Multivariate Approach

**DOI:** 10.1371/journal.pone.0153772

**Published:** 2016-04-18

**Authors:** Bárbara Oliveiros, Mafalda Sanches, Bruno Quendera, Bruno Graça, Daniela Guelho, Leonor Gomes, Francisco Carrilho, Filipe Caseiro-Alves, Miguel Castelo-Branco

**Affiliations:** 1 Laboratory of Biostatistics and Medical Informatics, Faculty of Medicine, University of Coimbra, Coimbra, Portugal; 2 Institute of Nuclear Sciences Applied to Health, Faculty of Medicine, University of Coimbra, Coimbra, Portugal; 3 Medical Imaging Department, University Centre Hospitals of Coimbra, Coimbra, Portugal; 4 Endocrinology, Diabetes and Metabolism Department, University Centre Hospitals of Coimbra, Coimbra, Portugal; 5 Institute for Biomedical Imaging and Life Sciences, University of Coimbra, Coimbra, Portugal; National Institutes of Health, UNITED STATES

## Abstract

Cardiovascular disease and diabetes represent a major public health concern. The former is the most frequent cause of death and disability in patients with type 2 diabetes, where left ventricular dysfunction is highly prevalent. Moreover, diabetic retinopathy is becoming a dominant cause of visual impairment and blindness. The complex relation between cardiovascular disease and diabetic retinopathy as a function of ageing, obesity and hypertension remains to be clarified. Here, we investigated such relations in patients with diabetes type 2, in subjects with neither overt heart disease nor advanced proliferative diabetic retinopathy. We studied 47 patients and 50 controls, aged between 45 and 65 years, equally distributed according to gender. From the 36 measures regarding visual structure and function, and the 11 measures concerning left ventricle function, we performed data reduction to obtain eight new derived variables, seven of which related to the eye, adjusted for age, gender, body mass index and high blood pressure using both discriminant analysis (DA) and logistic regression (LR). We found moderate to strong correlation between left ventricle function and the eye constructs: minimum correlation was found for psychophysical motion thresholds (DA: 0.734; LR: 0.666), while the maximum correlation was achieved with structural volume density in the neural retina (DA: 0.786; LR: 0.788). Controlling the effect of pairwise correlated visual constructs, the parameters that were most correlated to left ventricle function were volume density in retina and thickness of the retinal nerve fiber layers (adjusted multiple R^2^ is 0.819 and 0.730 for DA and LR), with additional contribution of psychophysical loss in achromatic contrast discrimination. We conclude that visual structural and functional changes in type 2 diabetes are related to heart dysfunction, when the effects of clinical, demographic and associated risk factors are taken into account, revealing a genuine relation between cardiac and retinal diabetic phenotypes.

## Introduction

Diabetes is a major cause of morbidity and mortality due to its multiorgan complications [1–3]. In particular, the incidence of type 2 diabetes is increasing, as well as its burden, mainly due to population ageing, but also as a result of alterations in lifestyles, which are leading to the reduction of physical activity and to the increase of obesity [1–3].

With the increase of diabetes in the population, diabetic retinopathy was added to the priority list of preventable visual impairment, since it affects about 4.25% of the world population, and highly contributes to low vision estimates around the world [4, 5].

Moreover, diabetes is related to other major organ complications, in particular nephropathy, which is the leading cause of kidney failure and most importantly cardiac disease (in fact about half of diabetics die from cardiovascular disease). This is due to the fact that type 2 diabetes mellitus is reflected in increased coronary atherosclerosis and left ventricular dysfunction [5, 6], conditions that are highly prevalent in this condition. According to the World Health Organization, cardiovascular disease is the most frequent cause of death and disability in patients with type 2 diabetes[2].

Metabolic and physiological risk factors known to be associated with the increase of these deaths are raised blood pressure, being overweight or obese, hyperglycaemia and hyperlipidaemia.

According to the Annual Report from a National Observatory of Diabetes from 2012, the prevalence of diabetes in southern European populations can reach 12.7%, although more than half remain undiagnosed. According to that report, attributable deaths due to diabetes were 4.4% of the total deaths, cardiovascular diseases being a major cause of death [7].

Previous studies addressing the relationship between diabetic retinopathy and heart complications have been largely univariate and/or included patients in relatively advanced stages [8–11]. Here we aimed to understand the relation between these complication types, in light of the complex multivariate profile that is typical of these patients.

Our aim was therefore to establish a relationship between structural alterations in retina, as measured by morphometric analysis, and functional measures, based on motion, colour and/or contrast discrimination with left ventricular alterations measured by cardiovascular magnetic resonance, as a function of other general clinical variables.

## Materials and Methods

### Protocol

This study was designed as a pilot, observational and prospective study with one visit where controls and type 2 diabetes mellitus patients performed multimodal imaging examinations, namely, ophthalmological, psychophysical, heart, liver and cerebral imaging. All subjects included in this study were evaluated for eligibility and signed the informed consent, approved by the Ethics Committee from the University of Coimbra. Our institutional ethics committee specifically approved this study.

Inclusion criteria were as follows: men and women aged between 40 and 75 years, functionally independent, capable to provide written consent after proper education and discussion with the treating physician and/or the research physician, with type 2 diabetes for the diabetic group and without any type of diabetes for the control group. Exclusion criteria defined for the study were: history of neuropsychiatric, renal, heart, ocular or any other severe non-age disease unrelated to diabetes, pregnancy or lactation. Eligible patients were asked to participate in the study and signed the informed consent form. Then, subjects completed a questionnaire on lifestyle, cardiovascular risk factors and family history of diabetes, current medication, with a particular emphasis in determining the presence or absence of medication for controlling arterial hypertension, physical activity, dietary pattern and quality of life.

Afterwards, at the clinic, height, weight, waist perimeter and blood pressure were measured. For the purpose of analysis, height and weight were used to derive body mass index, and waist was discarded as it was collected and registered in few cases. Clinically significant high blood pressure was defined by the current use (presence or absence) of medication for controlling arterial blood pressure.

All subjects performed ophthalmic examination including best corrected visual acuity and intraocular pressure measurements, visual psychophysical tests (Speed, Colour and Contrast Discrimination) and multimodal imaging (cerebral, heart, liver and ophthalmological scanning).

For the purpose of this particular study, we considered just heart and ophthalmological scanning. Concerning heart imaging, we used True fast imaging with steady state precision (True-FISP) obtained for the heart, performed at 3.0 T (Magnetom Trio; Siemens, Erlanger, Germany). The participants performed the True-FISP imaging in the supine position, with breath-hold at the end of expiration for each image acquisition, in order to discard artefacts due to respiratory motion. Measures were obtained for systolic and diastolic volume (SV and DV, mL), and the correspondent percentage of ejection fraction, weight of the left ventricle (WLV, g), left ventricle peak flow rate (PFR, mL/s), mitral peak E and peak A velocity (PE and PA, cm/s) as well as the mitral E/A ratio, mitral deceleration time (MDT, ms) and minimum and maximum left atrium volume (LAMIN and LAMAX, mL).

Optical Coherence Tomography (OCT) became an integral part of retinal clinical practice due to its detailed visualization of the retina morphology [12, 13]. OCT was performed for both eyes with the Frequency Domain Spectralis OCT (Heidelberg Engineering, Heidelberg, Germany). We measured both Volume Scan density (OCT VS) and Retinal Nerve Fiber Layer thickness (OCT RNFL). Volume scan density, in micrometres (μm), was acquired for the central subfield (CS), within 1mm of the centre of the macula; volume scan density was also acquired for nasal, temporal, superior and inferior quadrants either in the inner region (IN, IT, IS, II), within 1 and 3 mm of the centre of the macula, and in the outer region comprised between 3 and 6 mm of the centre of the macula (ON, OT, OS, OI). The retinal nerve fiber layers thickness, in micrometres (μm), was obtained for nasal and temporal quadrants (N, T) and for nasal superior (NS), nasal inferior (NI), temporal superior (TS) and temporal inferior (TI) regions, obtained within π mm of the centre of the macula. A global measure was also obtained (G).

Computerized psychophysical tests from the multifunctional module of the threshold of visual discrimination measure the ability of subjects to detect movement (Speed test, after abbreviated as SPEED), achromatic contrast (Achromatic test, after abbreviated as ACHROM) and chromatic contrast according to Protan, Deutan and Tritan axes, corresponding to distinct cone populations (after abbreviated as CHROM-P, CHROM-D and CHROM-T). All the tests used lateral, randomly moving pairs of dots, one being the reference point within each meridian used. Peripheral presented stimuli were of short duration, between 400 and 900 milliseconds, and also of short dimension and reduced spatial amplitude (about 1 degree of the visual angle). Periphery distances to the fixation point were of 7.5 visual degrees if the selected meridian was the 0° or 90°, or of 10 or 15 visual degrees on meridians 45° and 135°, respectively. Central fixation was controlled by an eye-tracker device, and that information was used in real time to validate the trial. If there was no central fixation, the trial would be successively repeated until validation. Response to each trial was given after a sound stimulus, which occurred at the end of vertical fixation. Properties as screen background point size and central cross remained constant for all the tests (speed, achromatic and chromatic), and the only property (dependent variable) that changed was the one being analysed at each case. These tests returned a threshold that represents the minimal difference between the properties being analysed to the asymptotic value at chance level. The screen background was achromatic (grey), and the luminance used had a sufficient magnitude to guarantee that the test occurred in conditions of photopic response (30 candelas/m^2^).

As visual psychophysical tests were performed just for the dominant eye, we selected just the dominant’s eye for analysis in all OCT measures, since data from both eyes are highly correlated and the measure of interest is the subject, not the eye [14, 15].

### Sample description

Ninety seven subjects (51 males and 46 females) aged between 45 and 65 years old (54.67 ± 0.65 years) with mean body mass index of 27.09 ± 0.40 kg/m^2^ (range between 18.50 and 34.90 kg/m^2^), fulfilling the inclusion/exclusion criteria for this study were included in the analysis.

These subjects were classified according to the absence or presence of type 2 diabetes, according to the general gold standard for diagnosis. Thus, 50 subjects were controls for this disease (51.55%), and the remaining 47 subjects were type 2 diabetics (48%), with non-proliferative diabetic retinopathy. None of the subjects had chronic eye or heart disease; nevertheless, 41 of the 97 subjects (42.27%) were undergoing therapy for hypertension.

### Statistical Analysis

All qualitative data are expressed in terms of counts and observed percentages. For all quantitative data we present its range, mean and standard error of the mean, and also its median values and the 25^th^ and 75^th^ percentiles. Normality was assessed through the Kolmogorov-Smirnov test in order to decide whether to use Pearson or Spearman rank-order correlation coefficient, or to decide between Student’s t test and Mann-Whitney’s test for independent univariate comparisons.

As there were 50 variables in the analysis, for less than 100 participants, and multiple comparisons problem would arise between correlated variables, new (derived) variables were generated using two methods applied in this context to perform data reduction: discriminant analysis and logistic regression, considering the presence of type 2 diabetes mellitus as dependent variable. In this particular case, both were used in order to perform a variable reduction, generating an overall measure for each set of variables. The motive for using two different methods was for confirmatory reasons. This is because some the assumptions of discriminant analysis might not hold [16, 17]. The advantage of logistic regression to discriminant analysis is the lack of assumptions about normality and homogeneity of variance matrices [18, 19], and may be used as a confirmatory analysis instead of using structural equation modelling, which would involve similar assumptions as discriminant analysis.

We took into account that diabetes and cardiovascular disease prevalence are dependent on age, gender, body mass index and the presence of arterial hypertension. Therefore, this set of variables was forced to enter in all the variable reduction that was performed.

Variable reduction with these methods permitted us to obtain new constructs related to each set of structural and functional assessment (OCT VS, OCT RNFL, SPEED, ACHROM, CHROM-P, CHROM-D, CHROM-T, True-FISP), and work with 8 variables (f_1_ to f_8_, described below), summarizing, with correction, structural integrity of the retina (1 and 2), visual (3–7) and cardiac function (8) for 97 participants instead of 50 variables for 97 participants, each one of them corrected for age, gender, body mass index and diagnosed hypertension. These are depicted as follows, where non-identified abbreviations are the individual measurements described for each construct within the Protocol sub-section:

1. OCT VS = f_1_(HBP, gender, age, BMI, CS, IN, IS, IT, II, ON, OS, OT, OI)
2. OCT RNFL = f_2_(HBP, gender, age, BMI, N, NS, NI, T, TS, TI)
3. SPEED = f_3_(HBP, gender, age, BMI, S0°, S45°, S90°, S135°)
4. ACHROM = f_4_(HBP, gender, age, BMI, A0°, A45°, A90°, A135°)
5. CHROM-P = f_5_(HBP, gender, age, BMI, P0°, P45°, P90°, P135°)
6. CHROM-D = f_6_(HBP, gender, age, BMI, D0°, D45°, D90°, D135°)
7. CHROM-T = f_7_(HBP, gender, age, BMI, T0°, T45°, T90°, T135°)
8. True-FISP = f_8_(HBP, gender, age, BMI, DV, SV, WVE, PFR, PE, PA, MDT, LAMIN, LAMAX)

For the true FISP construct (variable 8), and in spite of having other variables such as the ejection fraction and the ratio between Peak E and Peak A, they were not considered as they are function, respectively, of systolic and diastolic volume (EF (%) = 100-SV/DV*100), and values for peak E and peak A (EA = peak E/peak A). Each function f_j_ with j from 1 to 8, was obtained either by Discriminant Analysis (functions DA_1_ to DA_8_) and by Logistic regression (functions LR_1_ to LR_8_).

Logistic regression delivers a probability, for each one of the eight constructs, of a participant to have type 2 diabetes, given the set of independent characteristics involved, and may be written as ${LR}_{j}\;=\;P\left(D\backslash X_{1},\ldots ,X_{p_{j}}\right)=\frac{e^{b_{0}+\sum_{i=1}^{p_{j}}b_{i}X_{i}}}{1+e^{b_{0}+\sum_{i=1}^{p_{j}}b_{i}X_{i}}},\;j=\bar{1,8}$, where p_j_ is the number of variables used for each construct.

Discriminant analysis delivers linear functions based on the discriminant scores (DS) obtained and given by each one of the eight linear discriminant functions defined as ${DS}_{j}\;=b_{0}+\sum_{i=1}^{p_{j}}b_{i}X_{i},\;j=\bar{1,8}$ where p_j_ is the number of variables used for each construct. The right probability of significance for the chi-square distribution of the squared Mahanabolis distances (D^2^) [17] between the score obtained in the discriminant function (*DS*_*j*_), and the centroid ($\bar{d_{j}}$) for the type 2 diabetic group, with one degree of freedom, is the intended function for the probability defined as ${DA}_{j}=P\left(D\backslash X_{1},\ldots ,X_{p_{j}}\right)=P(\chi_{1}^{2}>D^{2})$. We will use this probability instead of discriminant linear functions, which would be simpler, in order to obtain a comparable measure to logistic regression.

Both discriminant analysis and logistic regression procedures were performed using the *Enter* method, in order to force models to use age, gender, body mass index and presence of hypertension, and also all the variables involved in each construct, unless measured variables were linear combinations of others which would create multicollinearity and over-fitting.

Therefore, each construct represented the conditional probability of a participant to have type 2 diabetes, given a set of independent measures, and it would be a measure of positive correlation with type 2 diabetes given a specific set of variables.

Receiver Operating characteristic curves were obtained in order to validate these constructs, since they may only measure positive correlation between the presence of type 2 diabetes and each construct if they discriminate between groups, that is, if they may be used as classifiers. Also canonical correlation and Wilk’s statistic for discriminant analysis and Nagelkerke’s R^2^ and Hosmer and Lemeshow test for logistic regression were presented, in order to validate models and measure their fitness.

Finally, correlation between eye-related constructs and left ventricle impairment construct, were obtained. Afterwards, stepwise multiple linear regression using true-FISP as dependent variable, and eye-related constructs as independent was performed in order to eliminate multicollinearity between eye-related derived variables. This procedure enabled the identification of correlation, moderated by the probability of the presence of type 2 diabetes, between the heart-related construct and a linear combination of eye-related constructs, using age, gender, body mass index and blood pressure as covariates.

Statistical analysis was performed using SPSS®, version 22, and all statistical tests were evaluated at a 5% significance level. Confidence intervals (95%) are presented for area under the ROC curve.

## Results

### Univariate predictors of Type 2 diabetes mellitus

#### Clinical and sociodemographic information

The sample was gender-matched but, as expected, type 2 diabetes participants presented higher prevalence or diagnosed hypertension (Table 1), since 68.09% of all type 2 diabetic participants were undergoing therapy for raised blood pressure, in opposition to 18.00% of subjects with arterial hypertension in the control group.

**Table 1:** Descriptive statistics for qualitative sociodemographic data and comparison between types of participants.

		Control			Diabetic			STS (p-value)
		Count	%\type	%\variable	Count	%\type	%\variable	
Gender	M	24	48.00%	47.06%	27	57.45%	52.94%	0.87 (0.352)
	F	26	52.00%	56.52%	20	42.55%	43.48%	
Blood Pressure	HBP	9	18.00%	21.95%	32	68.09%	78.05%	24.91 (< 0.001)
	NHP	41	82.00%	73.21%	15	31.91%	26.79%	

HBP–High Blood Pressure (subjects with previous diagnose of Arterial Hypertension, and undergoing therapy for that disease); NBP–Normal Blood Pressure (subjects without previous diagnostic of hypertension); STS–Standardized Test Statistic (*χ*2); %\type is the percentage relative to the total number of controls or diabetic participants, whether %\variable is the percentage relative to the total number of males or females for variable gender, and relative to the total number of hypertensive or normotensive participants for variable blood pressure.

Controls were also younger and presented lower body mass index than type 2 diabetics (Table 2); in fact, type 2 diabetic participants were showed to have a mean BMI more than 3.01 kg/m^2^ than controls. However, note that both groups presented mean values for body mass index within overweight limits. Moreover, note that derived variables were corrected for gender, blood pressure, and age and body mass index.

**Table 2:** Descriptive statistics for quantitative sociodemographic data and comparison according to Type of participants.

	Group	Count	Min	Max	Mean	SEM	P25	Median	P75	STS (p-value)
Age	Control	50	45.00	65.00	52.46	0.89	48.00	50.00	57.00	-3.52** (< 0.001)
(years)	Diabetic	47	45.00	65.00	57.02	0.85	52.00	58.00	62.00	
Body Mass Index	Control	50	18.50	33.40	25.63	0.45	23.90	24.90	27.80	-3.98* (p < 0.001)
(kg/m^2^)	Diabetic	47	20.50	34.90	28.64	0.61	25.60	28.70	33.10	

Min–minimum; Max–maximum; SEM–standard error of the mean; P25 –percentile 25; P75 –percentile 75; STS–Standardized Test Statistic obtained for the independent samples t- test (*) or for the Mann-Whitney U test (**)

#### Measures of retinal structure and function in T2DM

Concerning eye related variables measured with OCT, we only found statistically significant differences between the two types of participants on the temporal-inferior region of the Retinal Nerve Fiber Layer (STS = 2.56; p = 0.012), where type 2 diabetic participants presented thinner retinal nerve fiber layers. There was also a tendency for a thinning on the retinal nerve fiber layer in the nonproliferative type 2 diabetic group on the nasal superior region of the dominant eye (STS = 1.96; p = 0.053). These results might be explained by age differences, thus the need for multivariate analysis. Further analysis of univariate results is presented in S1 Table.

We found that diabetic participants presented lower motion or movement detection discrimination under meridians 0° and 135° (respectively STS = -2.51; p = 0.012 and STS = -2.91; p = 0.004) than controls (S2 Table). Concerning contrast sensitivity, type 2 diabetic patients seemed to show worse achromatic contrast sensitivity along meridian 90° (STS = -2.95; p = 0.004), and worse chromatic contrast sensitivity along meridian 0° of the Protan axis (STS = -2.00; p = 0.046) and Deutan axis (STS = -2.80; p = 0.005), although we found, also, a tangentially significant difference on meridian 90° of Deutan axis (STS = -1.91; p = 0.056) of chromatic cones. Concerning the Tritan axis of chromatic contrast sensitivity, this axis may have higher discriminant power between groups, since it presented statistically significant differences along all the four measured meridians (S2 Table).

#### Left ventricular dysfunction in T2DM

Type 2 diabetic patients seemed to show lower Systolic and Diastolic volume (respectively STS = -2.49; p = 0.013 and STS = 2-.62; p = 0.009), despite no statistically significant differences being found in the percent of Eject Fraction (STS = -0.17; p = 0.868). Although Peak E of controls and type 2 diabetics was similar (STS = -0.60; p = 0.551), diabetic participants presented statistically significant higher values of Peak A (STS -2.55; p = 0.011) and, thus significantly lower values on the ratio between peaks E and A (STS = -2.86; p = 0.004), as presented in S3 Table.

### Data reduction and validation of new derived variables—Eye and Heart related constructs

The other variables did not show significant statistical differences between groups (as presented in S1 Table to S3 Table), but they might have some extra contribution to sum into the derived variables, thus they were forced to be included in each one of their related constructs, unless they were a linear combination of other original variables, such as the percent of ejection fraction and the ratio between peak E and peak A. For each method used to create constructs, eight functions were obtained, always considering age, gender, blood pressure controlled by medication and body mass index as covariates, either with discriminant analysis (DA) or logistic regression (LR).

Both methods for variable reduction and derivation of the new variables for the constructs produced effective results for prediction of type 2 diabetes based on the probability of having diabetes, given sociodemographics and each characteristic analysed (ophthalmological/psychophysical or left ventricle dysfunction, evaluated by True-FISP). This result is a measure of accuracy of each construct.

Wilk’s Lamda test (Table 1) indicates a statistically significant relationship between the independents entered into each discriminant analysis model and the dependent variable, that is, the construct that is being derived, and thus indirectly measures the goodness of fit of those models. The Hosmer and Lemeshow test (HL) indicates the goodness of fit of each logistic regression model derived for each one of the eight constructs (Table 3). In this table, we may also observe that the minimal canonical correlation between independents and the dependent variable on DA models was obtained for chromatic contrast discrimination along the Protan axis (0.624), and the minimum value for the Nagelkerke R^2^ is obtained for the same construct (R^2^ = 0.536). Both of those measures showed to be, at least, reasonable, and the obtained correspondent values for the all the other derived variables were higher.

**Table 3:** Validation summary for constructs derived from original variables.

Construct	Method	Canonical correlation	Wilks' *λ* / HL				R^2^ Nagelkerke	ROC				
			Wilks' *λ*	*χ*^2^	df	p-value		AUC	SE	p-value	LB95%	UB95%
VS	DA	0.641	0.59	46.86	13	< 0.001	-	0.874	0.04	< 0.001	0.797	0.951
	LR	-	HL	2.79	8	0.947	0.560	0.877	0.04	< 0.001	0.801	0.952
RNFL	DA	0.652	0.58	49.50	11	< 0.001	-	0.876	0.04	< 0.001	0.799	0.952
	LR	-	HL	3.40	8	0.907	0.563	0.874	0.04	< 0.001	0.796	0.951
SPEED	DA	0.656	0.57	51.23	8	< 0.001	-	0.904	0.03	< 0.001	0.836	0.972
	LR	-	HL	8.81	8	0.358	0.593	0.922	0.03	< 0.001	0.862	0.981
ACHROM	DA	0.636	0.60	47.12	8	< 0.001	-	0.888	0.04	< 0.001	0.815	0.962
	LR	-	HL	8.52	8	0.384	0.537	0.888	0.04	< 0.001	0.814	0.961
CHROM-	DA	0.624	0.61	44.98	8	< 0.001	-	0.844	0.05	< 0.001	0.755	0.933
P	LR	-	HL	9.68	8	0.288	0.536	0.845	0.05	< 0.001	0.754	0.935
CHROM-	DA	0.628	0.61	45.71	8	< 0.001	-	0.847	0.04	< 0.001	0.762	0.933
D	LR	-	HL	3.60	8	0.891	0.546	0.847	0.04	< 0.001	0.760	0.934
CHROM-	DA	0.661	0.56	52.31	8	< 0.001	-	0.874	0.04	< 0.001	0.793	0.956
T	LR	-	HL	6.10	8	0.636	0.580	0.879	0.04	< 0.001	0.801	0.958
True-FISP	DA	0.684	0.53	55.89	13	< 0.001	-	0.940	0.03	< 0.001	0.882	0.999
	LR	-	HL	3.08	8	0.929	0.647	0.958	0.02	< 0.001	0.911	1.000

HL, Hosmer and Lemeshow Test

Besides, the areas under the ROC curve presented excellent discrimination between type 2 diabetics and controls, being able to separate groups with an accuracy of, at least, 84.4% (p < 0.001) with DA and 84.5% (p < 0.001) with LR methods (Table 3). These values were obtained for chromatic contrast sensitivity along the Protan axis, in concordance with canonical correlation and Nagelkerke’s R^2^. All the other constructs presented higher area under the ROC curve, whichever the method applied.

Therefore, these eight constructs might be considered as validated to be used as new variables, and we may be able to trace the type 2 diabetes profile based on each construct, according to the obtained coefficients for each method, presented on S4 Table. Using the volume scan density, we may say that a subject with diagnosed hypertension and undergoing treatment for that disease, female, slightly older, with increase body mass index and increased density on the outer region of the macula, and also on the inner superior region, seems to have higher probability of being a type 2 diabetes patient, according to the DA model. The LR model does not consider the outer inferior region of the eye as an increasing risk factor for type 2 diabetes, as it has a negative coefficient.

In fact, DA and LR models are quite concordant about signals of their coefficients, which are translated in an increase higher risk for the presence of type 2 diabetes whenever they are positive. Dissimilarities are observed for the Outer Inferior region of volume scan density, as referred before, and for variables gender and thickness on the nasal region for the RNFL construct, and for variables Mitral Deceleration Time and maximum volume obtained for Left Atrium in what concerns the True-FISP construct.

### Type 2 diabetic patient profiling based on eye and heart phenotype

In fact, DA and LR use different weights for each variable within a set (S4 Table), but both define the profile of a type 2 diabetic subject as one that presents higher volume density at the inner superior, outer nasal, superior and temporal regions of retina, higher thickness of the retinal nerve fiber layer at the temporal region, as also for its global value, with less perception of movement on the 0° and 135° and less achromatic contrast along the 0°, 45° and 90° meridians, and worse chromatic contrast sensitivity whichever the axes considered (Protan, Deutan or Tritan), at least in three out of the four meridians measured, giving a special emphasis on the 0° meridian. In what concerns left ventricle measures, type 2 diabetes mellitus subjects were more probable to have increased systolic volume, mitral peak A velocity and left atrium volume with decreased diastolic volume, weight of the left ventricle, mitral peak E velocity and deceleration time, when compared to controls.

### Correlation between the Neuro-Retinal Phenotype and Left Ventricular dysfunction

**Fig 1:**
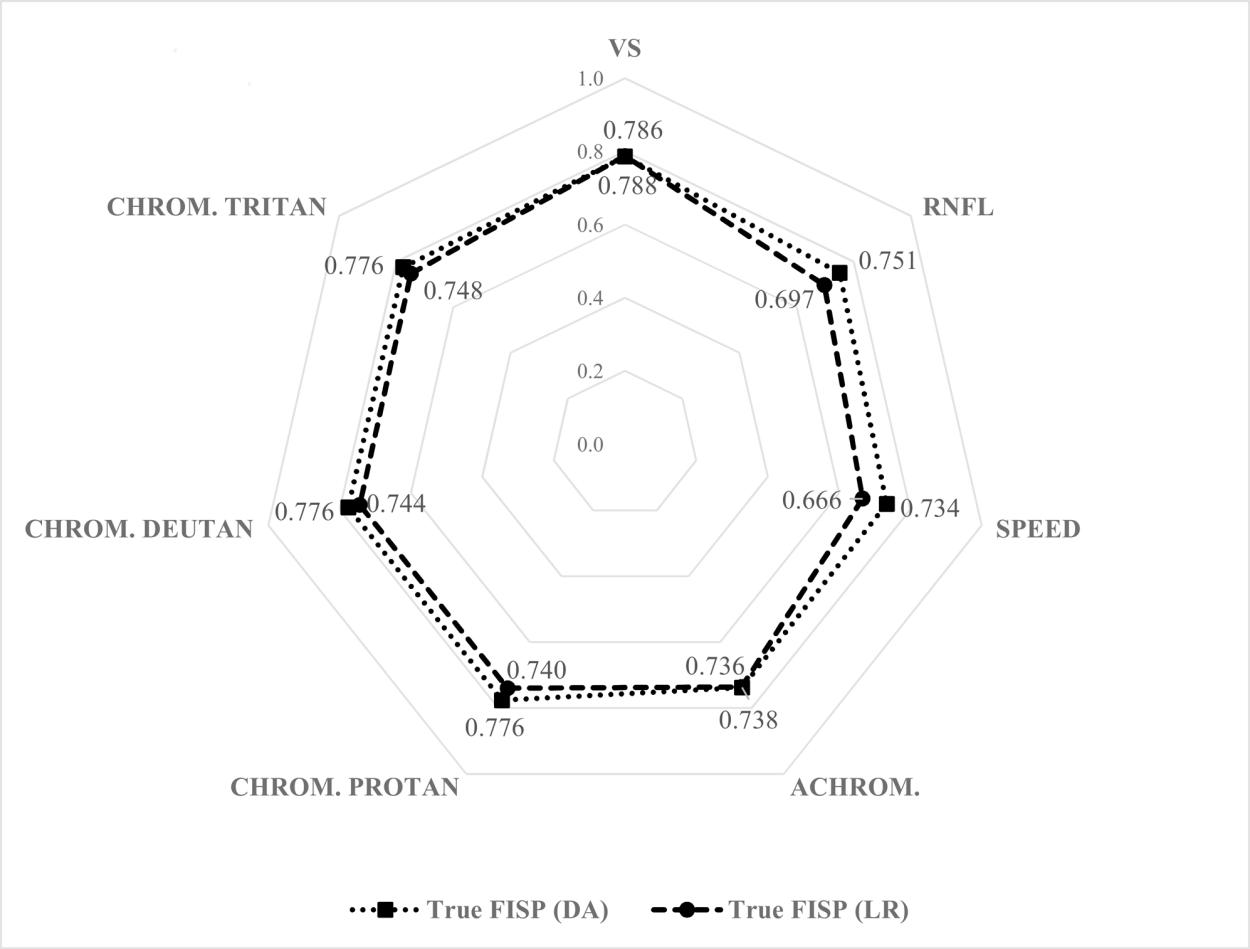
Correlation coefficient between True-FISP and each one of the new eye related constructs. All correlation coefficients were determined in the new derived variables, obtained either with DA or LR method, adjusted for age, gender, body mass index and blood pressure., obtained with DA and LR methods. The Spearman coefficient is presented, for each method, nearby the construct.

Whichever the method used, the new eye-related constructs obtained, adjusted for gender, age, blood pressure and body mass index appeared to be strongly correlated with the new left ventricle related construct, also adjusted for the same set of variables, as we may observe in Fig 1. The smallest correlation coefficient is obtained between True-FISP and Speed, whichever the method applied (DA: r = 0.734; LR: r = 0.666), and the highest correlation is measured between True-FISP and volume scan density (DA: r = 0.786; LR: r = 0.788). With the sample size that is being used, even the smaller correlation coefficient presents high statistical significance (p < 0.001).

However, these variables are, obviously, correlated between themselves thus stepwise multiple regression may identify which is the minimum set of eye-related constructs that are statistically correlated with heart-related construct, all adjusted for gender, age, body mass index and blood pressure. Both models presented acceptable Durbin-Watson statistic (DA: 1.93; LR: 1.84), showing that residuals from this regression are serially uncorrelated.

The Discriminant Analysis method for variable reduction leads to a solution which considers that variability in Volume Scan (p = 0.003), Retinal Nerve Fibre Layer (p = 0.014) and Achromatic contrast (p = 0.020) constructs explain 81.9% of the variability obtained in True FISP construct, while the Logistic Regression method only considers the same first two variables in the model, VS (p < 0.001) and RNFL (p = 0.001), with an adjusted R^2^ of 0.730 (Table 4). However, if another variable was to be included into the model, it would be achromatic contrast discrimination since it is the one with smallest p-value within the excluded variables (p = 0.098).

**Table 4:** Independently correlated eye-related constructs and left ventricular function.

Model	Constructs	R	R^2^	Adjusted R^2^	Durbin-Watson	F	df	p-value
DA	VS + RNFL + ACHROM	0.908	0.825	0.819	1.925	145.69	3, 93	< 0.001
LR	VS + RNFL	0.858	0.736	0.730	1.842	137.80	2, 94	< 0.001

df, degrees of freedom

**Fig 2:**
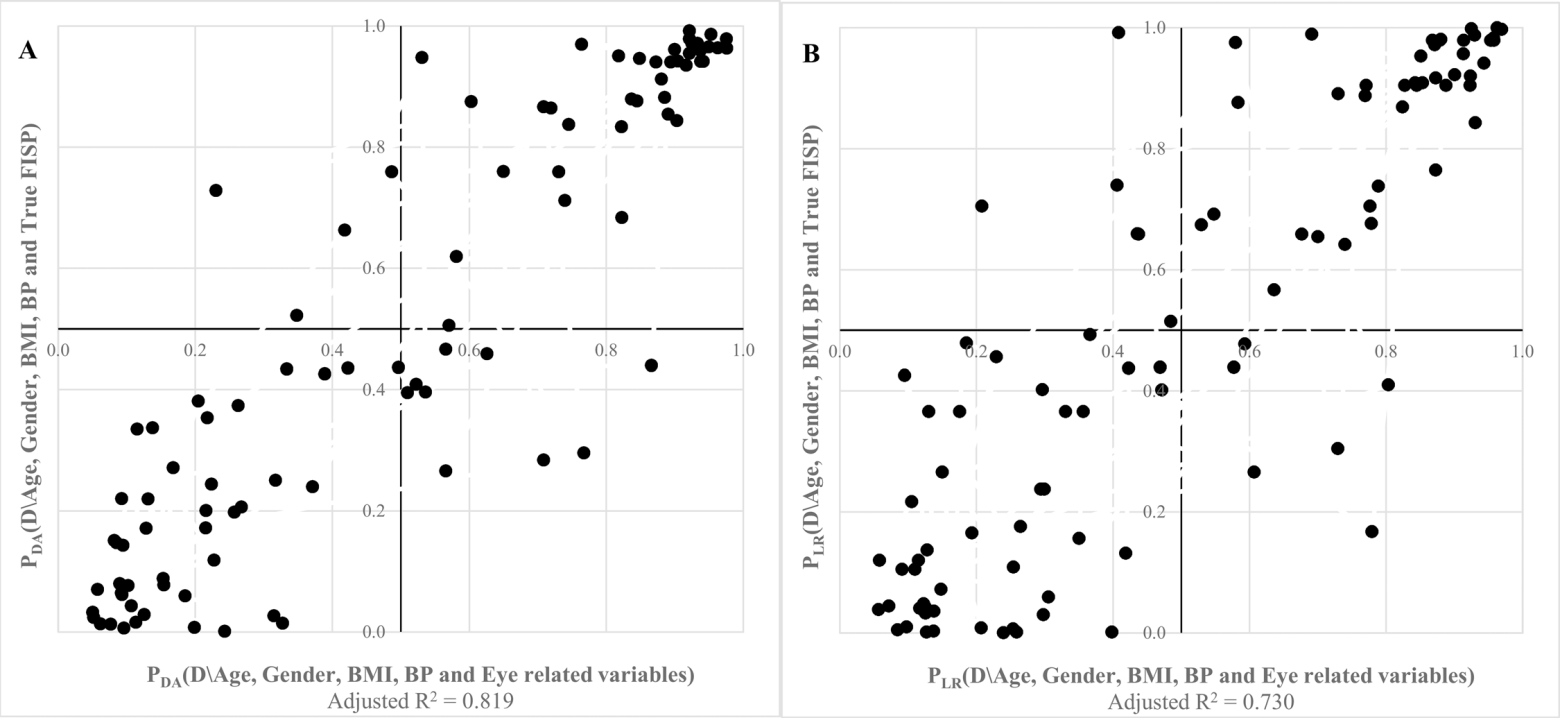
Correlation between eye and left ventricle function using discriminant analysis models (A) or logistic regression models (B).

In fact, correlation between the identified non-collinear sets of eye-related constructs measuring positive correlation to the presence of type 2 diabetes and the heart-related variables moderating the presence of type 2 diabetes present excellent correlation, as observed in table above (Table 4) and below in Fig 2, especially when probabilities for the presence of type 2 diabetes are simultaneously above 0.900 or when subjects are almost certainly controls for this disease. Using the DA model, standardized coefficients show that the stronger correlation is achieved with volume scan density (0.37; p = 0.003), followed by the thickness of retinal nerve fiber layer (2.50; p = 0.014) and achromatic contrast sensitivity (2.36; p = 0.20), which is concordant with the LR method that found standardized coefficients to be 0.48 (p < 0.001) for VS and 3.39 (p = 0.001) for RNFL, variables included into the model, and 0.33 (p = 0.098) for achromatic contrast.

**Fig 3:**
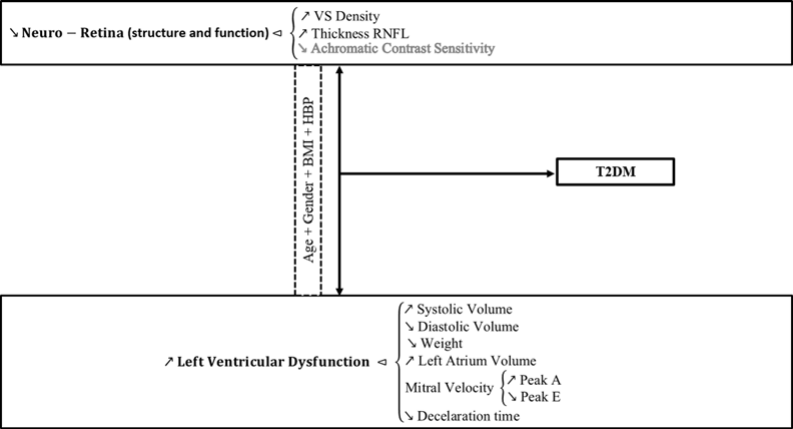
Graphical abstract highlighting our main results. Prediction of left ventricular dysfunction in type 2 diabetes (T2DM) based on independent visual dysfunction, moderated by common risk factors such as age, gender, body mass index (BMI) and presence/absence of drug controlled arterial hypertension (HBP).

Therefore, the loss of density in the neuro-retina and increased thickness of the retinal nerve fiber layers, naturally related to diabetic retinopathy and its impact on visual dysfunction, is correlated to left ventricle dysfunction in this type of patients. Such relation is described on Fig 3, showing that type 2 diabetes may be predicted either through visual or cardiac phenotype, both closely related when natural risk factors to type 2 diabetes are taken into account.

## Discussion

The main finding of this study was that early diabetic retinopathy and asymptomatic heart function are related, even when a large number of covariates are taken into account (see Fig 3 for Graphical Abstract highlighting the main findings).

Univariate approaches in analysing retinal and heart complications were in general less informative for two reasons: first, patients were in early stages or even asymptomatic. Second, correlations might be explained by common risk factors, which implies adjustment procedures and at the same time data reduction taking into account the variables that explain most of the variance. Most of the previous work has largely been based on such approaches. Kurioka *et al* [8] found an association between left ventricular dysfunction and both age and severity of diabetic retinopathy, classified into three groups (without diabetic retinopathy, nonproliferative diabetic retinopathy and pre or proliferative diabetic retinopathy). Moreover, he found that impaired left ventricular diastolic function was related with age and gender. This is the reason why in our study such parameters were used as covariates in the construction of each one of the new derived variables. Takeda *et al* [9] found that left ventricular diastolic dysfunction is associated with diabetic retinopathy, and its presence may explain part of the increased incidence of heart failure in diabetic patients, which is similar to state that diabetic retinopathy represents and increased risk of heart failure, independent from other known risk factors [10]. Given that common risk factors may explain such associations, it is important to parcel out common variance, as implemented here. The study published by Iris Walraven *et al* [11] is slightly different from the previous ones since it was a longitudinal study with eight years of follow-up. However, an association between left ventricular dysfunction in terms of lower ejection fraction and diabetic retinopathy was also found, although just in males, and no association was found in females or using other cardiac parameters. These findings are also consistent with common covariates, which we tried to take into account in the derived variables.

We therefore opted for a largely multivariate approach, coupled with derived constructs, that achieved data reduction and cross prediction between cardiac and visual structure/function variables. Interestingly, univariate statistical tests did not distinguish between type 2 diabetic participants and controls in what concerns volume scan density (S1 Table), which may be explained by the fact that patients were in an early disease stage. We only found statistically significant differences for retinal nerve fiber layers thickness, and only at the temporal inferior region, when covariates were not taken into account.

According to Andrade LC *et al* [20], luminance contrast sensitivity and colour vision are considered to have great predictive value in diabetic retinopathy. In fact, Roy *et al* [21] stated that type 1 diabetic patients have significantly more colour vision defects than controls, independently from age, gender, duration of the disease and its metabolic control, which was confirmed in a previous study from our group [22]; on the other hand, Wolf BE *et al* [23] recently claimed that colour vision is affected in type 2 diabetic patients, even when diabetic retinopathy is absent.

These studies suggest that colour contrast sensitivity decreases in presence of diabetes, but do not report most of the covariates used here. We also found that type 2 diabetic subjects presented lower contrast sensitivity in achromatic vision (S2 Table), at least along the meridian 90°, which may indicate a defect in the pathway underlying achromatic discrimination.

Colour contrast sensitivity is severely affected in type 2 diabetic patients, especially along the Tritan (blue cone) axis where we found significant statistical differences in contrast sensitivity whichever the meridian used, as presented before in Table 4. In fact, Tritan defects presented by type 2 diabetic subjects affect their short wavelength cones, affecting their vision along the blue/yellow axis, This characteristic is more evident than defects in Protan (red cone) and Deutan (green cone) axes which were more visible along meridian 0°.

Concerning heart related variables, namely left ventricle impairment as measured by cardiovascular magnetic resonance, it is known that left ventricular diastolic function is highly prevalent in type 2 diabetes [5, 24] and diastolic heart failure is a progressive disorder that may present even with normal ejection fraction [25, 26]. In fact, type 2 diabetic patients presented a similar index of ejection fraction (S3 Table) as controls, but significantly decreased values on diastolic volume and systolic volume when compared to controls, with normal and similar weight in the left ventricle. The analysis of the transmitral parameters showed that type 2 diabetics present identical mitral peak E velocity, when compared to controls, but significantly higher mitral peak E velocity which is translated in a lower mitral E/A ratio, which may indicate impaired myocardial relaxation and increased myocardial stiffness, thus eventually diastolic dysfunction [27].

For each set of variables, we always found some dependency between type 2 diabetes and visual or heart dysfunction, which was in general moderate when assessed at an univariate level. New constructs obtained for each set of variables, adjusted for age, gender, blood pressure and body mass index, using either discriminant analysis and or logistic regression, were able to classify and discriminate between groups, as presented in Table 3. In fact, both methods presented similar accuracy in prediction of type 2 diabetes, given by the areas under the ROC curves, and canonical correlation or Nagelkerke’s R^2^. The worst fit was obtained for chromatic vision along the Protan axis, whichever the statistical method used, but presenting good accuracy (minimum AUC between 0.844 and 0.845) and moderate to strong correlation between variables and the new defined construct, as it may be observed in Table 3. In fact, we may assume that either volume scan density of the retina, or retinal nerve fibre layer thickness, or speed sensitivity or even disabilities in rods and photoreceptors are shown to be correlated to the presence of type 2 diabetes as they discriminated the probability of its presence.

On the other hand, cardiovascular variables as assessed by MR imaging and corrected for age, gender, blood pressure and body mass index, acting as covariates, were also associated with type 2 diabetes presence.

Those new derived eye-related variables were revealed to be strongly correlated to the cardiovascular construct, as presented on Fig 1. In fact, obtained correlation coefficients between eye constructs and cardiovascular magnetic resonance generated with DA are between 0.734 and 0.786, as observed for motion thresholds, and Volume Scan density, respectively. Applying the LR method, minimum and maximum significant correlations were seen between the same pairs of variables as the ones measures with DA.

Simple partial correlations would be useful to ascertain which eye-related measures are associated with cardiovascular function in type 2 diabetes mellitus; however, it is difficult to control for the effect of six variables. An alternative method, which also assumes normality but is more robust to its absence, since it uses the F distribution, is multiple regression, where we may find which variables within the set of eye-related variables are most correlated to the heart-related construct, moderated by the presence of type 2 diabetes since every constructs were built in order to give the probability that a subject is a type 2 diabetic.

Therefore, by adjusting variables to age, gender, body mass index and blood pressure, we found that there exists a strong correlation between cardiovascular impairment and the thickness of the retinal nerve fiber layer and increase of volume scan density in retina, moderated by the presence of type 2 diabetes, as presented in Table 4, and that achromatic contrast sensitivity may also be associated to left ventricle impairment in type 2 diabetes. As seen in Fig 2 it is possible to observe that the thickness of the retinal nerve fiber layer and increase in density of retina volume linearly combined, considering also the decrease in achromatic contrast sensitivity, are positively correlated to left ventricle impairment in diabetes.

Therefore, visual dysfunction due to loss of density in the neuro-retina and increased thickness of the retinal nerve fiber layers, related to type 2 diabetes, is correlated to left ventricle dysfunction moderated by the presence of type 2 diabetes. Moreover, type 2 diabetes may either be accurately predicted through visual or by the cardiac phenotype.

We conclude that the cardiac and retinal diabetic phenotypes are related even when an array of clinical and demographic variables, and associated risk factors are taken into account. This means that a genuine mechanistic relation exists between diabetic retinopathy and cardiovascular complications.

## Supporting Information

S1 Table Descriptive statistics for OCT data (Volume Scan density and Retinal Nerve Fiber Layer) and comparison between types of participants. (DOCX)

S2 Table Descriptive statistics for Psychophysics (Speed, Achromatic and Chromatic Contrast Sensitivity) data and comparison between types of participants. (DOCX)

S3 Table Descriptive statistics for True Fast Imaging Steady State Procedures (True-FISP) and comparison between types of participants. (DOCX)

S4 Table DA and LR Model coefficients. (DOCX)

## Abbreviations

ACHROM: Computerized psychophysical test for visual discrimination of achromatic contrast
BMI: Body Mass Index
CHROM-D: Computerized psychophysical test for visual discrimination of chromatic contrast (Deutan axis)
CHROM-P: Computerized psychophysical test for visual discrimination of chromatic contrast (Protan axis)
CHROM-T: Computerized psychophysical test for visual discrimination of chromatic contrast (Tritan axis)
DA: Discriminant Analysis
DS: Discriminant Score obtained by Discriminant Analysis
HBP: High Blood Pressure (previously diagnosed and actually under treatment
LR: Logistic Regression
OCT: Optical Coherence Tomography
OCT RNFL: Retinal Nerve Fiber Layer Thickness obtained with Optical Coherence Tomography
OCT VS: Volume Scan density obtained with Optical Coherence Tomography
ROC: Receiver Operating Characteristic Curve
SPEED: Computerized psychophysical test for visual discrimination of movement detection
STS: Standardized Test Statistic
True-FISP: True Fast Imaging with Steady State Precision
