# Supplementary material for: Relations between Cardiac and Visual Phenotypes in Diabetes: A Multivariate Approach
Source: PLoS One. 2016 Apr 18;11(4):e0153772. doi: 10.1371/journal.pone.0153772 (PMC4835099; doi:10.1371/journal.pone.0153772)
Supplement: S1 Table — (DOCX) [file pone.0153772.s001.docx]

**S1 Table. Descriptive statistics for OCT data (Volume Scan density and Retinal Nerve Fiber Layer) and comparison between types of participants**

| Measure (Region) | Group | Count | Min | Max | Mean | SEM | P25 | Median | P75 | STS (p-value) |
| --- | --- | --- | --- | --- | --- | --- | --- | --- | --- | --- |
| VS (Central Subfield) | Control | 50 | 249.00 | 318.00 | 284.33 | 2.40 | 273.00 | 286.79 | 293.00 | -0.45^**^ (0.651) |
|  | Diabetic | 47 | 193.00 | 545.00 | 289.51 | 7.27 | 268.00 | 286.00 | 297.00 |  |
| VS (Inner Nasal) | Control | 50 | 313.00 | 388.00 | 347.33 | 2.09 | 339.00 | 346.22 | 355.00 | -0.65^**^ (0.513) |
|  | Diabetic | 47 | 236.00 | 511.00 | 345.77 | 5.65 | 338.00 | 346.22 | 356.00 |  |
| VS (Inner Superior) | Control | 50 | 250.00 | 379.00 | 342.91 | 2.74 | 336.00 | 345.35 | 353.00 | -0.70^**^ (0.483) |
|  | Diabetic | 47 | 249.00 | 519.00 | 346.17 | 5.16 | 336.00 | 345.00 | 351.00 |  |
| VS (Inner Temporal) | Control | 50 | 300.00 | 381.00 | 333.76 | 2.10 | 326.00 | 333.14 | 342.00 | -1.36^**^ (0.173) |
|  | Diabetic | 47 | 285.00 | 461.00 | 331.74 | 3.50 | 321.00 | 331.00 | 337.00 |  |
| VS (Inner Inferior) | Control | 50 | 313.00 | 384.00 | 341.50 | 2.13 | 334.00 | 339.57 | 353.00 | -1.24^**^ (0.216) |
|  | Diabetic | 47 | 261.00 | 427.00 | 337.82 | 4.03 | 325.00 | 339.00 | 343.00 |  |
| VS (Outer Nasal) | Control | 50 | 127.00 | 346.00 | 310.65 | 4.16 | 305.00 | 313.49 | 321.00 | -0.44^**^ (0.662) |
|  | Diabetic | 47 | 249.00 | 510.00 | 317.23 | 5.61 | 302.00 | 313.49 | 323.00 |  |
| VS (Outer Superior) | Control | 50 | 269.00 | 324.00 | 298.35 | 1.69 | 292.00 | 299.78 | 305.00 | -0.49^**^ (0.625) |
|  | Diabetic | 47 | 228.00 | 444.00 | 300.40 | 4.34 | 289.00 | 297.00 | 307.00 |  |
| VS (Outer Temporal) | Control | 50 | 252.00 | 317.00 | 285.89 | 1.95 | 276.00 | 287.66 | 294.00 | -1.17^**^ (0.243) |
|  | Diabetic | 47 | 247.00 | 396.00 | 285.76 | 3.38 | 273.00 | 285.00 | 291.00 |  |
| VS (Outer Inferior) | Control | 50 | 252.00 | 320.00 | 286.25 | 2.10 | 277.00 | 285.31 | 293.00 | -1.28^**^ (0.202) |
|  | Diabetic | 47 | 239.00 | 394.00 | 285.78 | 4.22 | 273.00 | 285.00 | 290.00 |  |
| RNFL (Global) | Control | 50 | 83.00 | 121.00 | 98.82 | 1.01 | 95.00 | 97.98 | 102.00 | -0.53^**^ (0.595) |
|  | Diabetic | 47 | 62.00 | 118.00 | 96.32 | 1.58 | 93.00 | 97.98 | 102.00 |  |
| RNFL (Nasal) | Control | 50 | 52.00 | 106.00 | 75.09 | 1.49 | 68.00 | 74.64 | 82.00 | 0.45^*^ (0.654) |
|  | Diabetic | 47 | 45.00 | 98.00 | 74.10 | 1.62 | 66.00 | 74.64 | 83.00 |  |
| RNFL (Nasal Superior) | Control | 50 | 67.00 | 151.00 | 108.74 | 2.94 | 92.00 | 102.86 | 124.00 | 1.96^*^ (0.053) |
|  | Diabetic | 47 | 54.00 | 153.00 | 100.11 | 3.30 | 82.00 | 102.86 | 116.00 |  |
| RNFL (Nasal Inferior) | Control | 50 | 83.00 | 168.00 | 120.57 | 2.96 | 104.00 | 119.50 | 135.00 | -0.37^**^ (0.715) |
|  | Diabetic | 47 | 59.00 | 162.00 | 119.82 | 3.06 | 105.00 | 121.08 | 137.00 |  |
| RNFL (Temporal) | Control | 50 | 51.00 | 97.00 | 68.50 | 1.31 | 62.00 | 70.00 | 72.00 | -0.53^*^ (0.597) |
|  | Diabetic | 47 | 32.00 | 95.00 | 69.65 | 1.78 | 64.00 | 71.54 | 77.00 |  |
| RNFL (Temporal Superior) | Control | 50 | 86.00 | 179.00 | 136.25 | 2.48 | 126.00 | 136.11 | 145.00 | 1.06^*^ (0.291) |
|  | Diabetic | 47 | 51.00 | 187.00 | 131.57 | 3.70 | 114.00 | 135.00 | 148.00 |  |
| RNFL (Temporal Inferior) | Control | 50 | 88.00 | 186.00 | 135.76 | 3.31 | 122.00 | 132.50 | 154.00 | 2.56^*^ (0.012) |
|  | Diabetic | 47 | 65.00 | 193.00 | 123.77 | 3.32 | 112.00 | 128.00 | 136.00 |  |

Min, minimum; Max, maximum; SEM, standard error of the mean; P25, percentile 25; P75, percentile 75; STS, Standardized Test Statistic obtained for the independent samples t- test (*) or for the Mann-Whitney U test (**)
