# Supplementary material for: Relations between Cardiac and Visual Phenotypes in Diabetes: A Multivariate Approach
Source: PLoS One. 2016 Apr 18;11(4):e0153772. doi: 10.1371/journal.pone.0153772 (PMC4835099; doi:10.1371/journal.pone.0153772)
Supplement: S2 Table — (DOCX) [file pone.0153772.s002.docx]

**S2 Table. Descriptive statistics for Psychophysics (Speed, Achromatic and Chromatic Contrast Sensitivity) data and comparison between types of participants**

| Measure (Meridian) | Group | Count | Min | Max | Mean | SEM | P25 | Median | P75 | STS (p-value) |
| --- | --- | --- | --- | --- | --- | --- | --- | --- | --- | --- |
| Speed, deg/s (0º) | Control | 50 | .16 | 3.00 | 1.17 | .09 | .68 | 1.04 | 1.63 | -2.51^**^ (0.012) |
|  | Diabetic | 47 | .17 | 9.69 | 1.96 | .28 | .75 | 1.63 | 2.23 |  |
| Speed, deg/s (45º) | Control | 50 | .16 | 7.46 | 1.45 | .18 | .56 | 1.28 | 1.75 | -0.50^**^ (0.615) |
|  | Diabetic | 47 | .15 | 21.44 | 1.81 | .45 | .64 | 1.24 | 1.82 |  |
| Speed, deg/s (90º) | Control | 50 | .16 | 8.39 | 1.42 | .18 | .74 | 1.16 | 1.66 | -1.12^**^ (0.264) |
|  | Diabetic | 47 | .32 | 9.77 | 1.75 | .25 | .77 | 1.24 | 2.05 |  |
| Speed, deg/s (135º) | Control | 50 | .15 | 7.13 | 1.29 | .16 | .58 | 1.03 | 1.82 | -2.91^**^ (0.004) |
|  | Diabetic | 47 | .15 | 20.40 | 2.62 | .47 | .68 | 1.86 | 2.91 |  |
| Achromatic, cd/m^2^ (0º) | Control | 50 | 1.00 | 5.44 | 2.62 | .13 | 1.93 | 2.60 | 3.10 | -0.70^**^ (0.481) |
|  | Diabetic | 47 | 1.00 | 10.47 | 2.66 | .23 | 1.51 | 2.57 | 3.03 |  |
| Achromatic, cd/m^2^ (45º) | Control | 50 | 1.00 | 5.75 | 2.56 | .14 | 1.93 | 2.32 | 2.85 | -1.12^**^ (0.262) |
|  | Diabetic | 47 | 1.00 | 7.03 | 2.79 | .18 | 1.93 | 2.83 | 3.10 |  |
| Achromatic, cd/m^2^ (90º) | Control | 50 | 1.00 | 4.85 | 2.34 | .14 | 1.51 | 2.31 | 2.73 | -2.95^*^ (0.004) |
|  | Diabetic | 47 | 1.00 | 5.92 | 3.00 | .17 | 2.21 | 2.73 | 3.66 |  |
| Achromatic, cd/m^2^ (135º) | Control | 50 | 1.20 | 5.64 | 2.66 | .13 | 2.01 | 2.60 | 3.20 | -0.22^**^ (0.823) |
|  | Diabetic | 47 | 1.00 | 5.85 | 2.64 | .16 | 2.00 | 2.65 | 3.10 |  |
| Chromatic Protan, x1000 (0º) | Control | 50 | 1.23 | 4.93 | 2.65 | .16 | 1.25 | 2.48 | 3.70 | -2.00^**^ (0.046) |
|  | Diabetic | 47 | 1.23 | 19.60 | 3.65 | .42 | 1.85 | 3.38 | 4.52 |  |
| Chromatic Protan, x1000 (45º) | Control | 50 | 1.23 | 28.63 | 6.04 | .70 | 2.49 | 4.83 | 8.23 | -0.30^**^ (0.767) |
|  | Diabetic | 47 | 1.23 | 35.21 | 7.13 | 1.02 | 2.47 | 6.54 | 8.03 |  |
| Chromatic Protan, x1000 (90º) | Control | 50 | 1.23 | 10.76 | 4.33 | .28 | 2.49 | 4.33 | 5.10 | -0.23^**^ (0.820) |
|  | Diabetic | 47 | 1.23 | 27.34 | 5.62 | .83 | 2.25 | 3.71 | 5.56 |  |
| Chromatic Protan, x1000 (135º) | Control | 50 | 1.24 | 30.23 | 6.31 | .66 | 4.11 | 5.53 | 7.42 | -0.71^**^ (0.476) |
|  | Diabetic | 47 | 1.23 | 18.39 | 5.71 | .51 | 3.49 | 5.72 | 6.79 |  |
| Chromatic Deutan, x1000 (0º) | Control | 50 | 1.23 | 9.15 | 3.46 | .28 | 1.85 | 3.08 | 4.93 | -2.8^**^ (0.005) |
|  | Diabetic | 47 | 1.24 | 55.95 | 8.17 | 1.83 | 2.48 | 4.33 | 6.62 |  |
| Chromatic Deutan, x1000 (45º) | Control | 50 | 1.24 | 83.52 | 21.17 | 3.28 | 3.71 | 10.39 | 27.48 | -1.44^**^ (0.150) |
|  | Diabetic | 47 | 1.24 | 76.32 | 24.70 | 3.00 | 7.08 | 24.61 | 28.86 |  |
| Chromatic Deutan, x1000 (90º) | Control | 50 | 1.23 | 47.47 | 8.13 | 1.16 | 3.08 | 5.48 | 12.46 | -1.91^**^ (0.056) |
|  | Diabetic | 47 | 1.23 | 76.95 | 15.44 | 2.62 | 3.71 | 10.47 | 15.89 |  |
| Chromatic Deutan, x1000 (135º) | Control | 50 | 1.23 | 72.73 | 16.06 | 2.14 | 5.56 | 13.22 | 19.71 | -1.28^**^ (0.201) |
|  | Diabetic | 47 | 1.23 | 69.83 | 21.11 | 2.69 | 6.82 | 18.37 | 24.99 |  |
| Chromatic Tritan, x1000 (0º) | Control | 50 | 14.99 | 111.62 | 48.08 | 2.84 | 30.45 | 44.16 | 56.81 | -3.57^**^ (< 0.001) |
|  | Diabetic | 47 | 25.00 | 427.31 | 101.48 | 15.05 | 43.33 | 67.44 | 99.35 |  |
| Chromatic Tritan, x1000 (45º) | Control | 50 | 25.00 | 305.13 | 102.77 | 9.41 | 48.83 | 87.01 | 128.47 | -2.33^**^ (0.020) |
|  | Diabetic | 47 | 30.23 | 374.24 | 130.22 | 10.83 | 66.96 | 128.47 | 145.99 |  |
| Chromatic Tritan, x1000 (90º) | Control | 50 | 28.33 | 237.92 | 68.58 | 5.41 | 40.00 | 65.61 | 87.42 | -3.02^**^ (0.003) |
|  | Diabetic | 47 | 25.00 | 425.51 | 111.73 | 13.00 | 56.81 | 78.57 | 108.45 |  |
| Chromatic Tritan, x1000 (135º) | Control | 50 | 14.99 | 263.37 | 89.75 | 7.48 | 46.66 | 78.08 | 120.33 | -2.60^**^ (0.009) |
|  | Diabetic | 47 | 35.23 | 568.07 | 142.25 | 16.11 | 65.84 | 120.33 | 170.97 |  |

Min, minimum; Max, maximum; SEM, standard error of the mean; P25, percentile 25; P75, percentile 75; STS, Standardized Test Statistic obtained for the independent samples t- test (*) or for the Mann-Whitney U test (**)
