# Supplementary material for: Relations between Cardiac and Visual Phenotypes in Diabetes: A Multivariate Approach
Source: PLoS One. 2016 Apr 18;11(4):e0153772. doi: 10.1371/journal.pone.0153772 (PMC4835099; doi:10.1371/journal.pone.0153772)
Supplement: S3 Table — (DOCX) [file pone.0153772.s003.docx]

**S3 Table. Descriptive statistics for True Fast Imaging Steady State Procedures (True-FISP) and comparison between types of participants**

| Measure | Group | Count | Min | Max | Mean | SEM | P25 | Median | P75 | STS (p-value) |
| --- | --- | --- | --- | --- | --- | --- | --- | --- | --- | --- |
| Eject Fraction (%) | Control | 50 | 56.00 | 76.00 | 66.55 | .52 | 65.00 | 66.68 | 67.00 | -0.17** (0.868) |
|  | Diabetic | 47 | 56.00 | 78.00 | 66.96 | .64 | 66.00 | 66.68 | 69.00 |  |
| Diastolic Volume (mL) | Control | 50 | 78.00 | 180.00 | 108.45 | 2.87 | 102.20 | 102.20 | 116.00 | -2.49** (0.013) |
|  | Diabetic | 47 | 65.00 | 133.00 | 97.73 | 1.89 | 93.00 | 102.20 | 102.20 |  |
| Systolic Volume (mL) | Control | 50 | 21.00 | 68.00 | 36.96 | 1.35 | 34.67 | 34.67 | 37.00 | -2.62** (0.009) |
|  | Diabetic | 47 | 18.00 | 55.00 | 32.74 | .99 | 30.00 | 34.67 | 34.67 |  |
| Weight Left Ventricule (g) | Control | 50 | 84.00 | 175.00 | 121.42 | 2.58 | 111.00 | 120.58 | 123.00 | -0.39** (0.694) |
|  | Diabetic | 47 | 73.00 | 207.00 | 120.02 | 3.22 | 115.00 | 120.58 | 120.58 |  |
| Peak Flow Rate (mL/s) | Control | 50 | 187.00 | 620.00 | 356.19 | 10.68 | 329.00 | 347.49 | 347.49 | -1.51** (0.132) |
|  | Diabetic | 47 | 155.00 | 494.00 | 325.66 | 8.60 | 300.00 | 347.49 | 347.49 |  |
| Mitral Peak E velocity (cm/s) | Control | 50 | 32.72 | 68.00 | 47.26 | 1.03 | 45.82 | 46.21 | 47.98 | -0.60** (0.551) |
|  | Diabetic | 47 | 30.00 | 61.88 | 45.62 | .95 | 43.70 | 46.21 | 47.35 |  |
| Mitral Peak A velocity (cm/s) | Control | 50 | 26.87 | 60.56 | 43.98 | .90 | 42.60 | 45.98 | 45.98 | -2.55** (0.011) |
|  | Diabetic | 47 | 33.38 | 74.76 | 47.44 | .95 | 45.98 | 45.98 | 48.71 |  |
| Mitral E/A ratio | Control | 50 | .68 | 2.10 | 1.12 | .03 | 1.06 | 1.06 | 1.18 | -2.86** (0.004) |
|  | Diabetic | 47 | .62 | 1.50 | 1.00 | .03 | .89 | 1.06 | 1.06 |  |
| Mitral Decelaration Time (ms) | Control | 50 | 110.00 | 290.00 | 177.77 | 4.23 | 169.00 | 175.03 | 180.00 | -0.69** (0.489) |
|  | Diabetic | 47 | 72.60 | 357.20 | 172.31 | 5.91 | 163.00 | 175.03 | 175.03 |  |
| Left Atrium minimum volume (mL) | Control | 50 | 13.81 | 53.78 | 31.25 | 1.05 | 28.50 | 31.76 | 31.76 | -0.32** (0.745) |
|  | Diabetic | 47 | 15.60 | 61.00 | 30.80 | 1.30 | 25.00 | 31.76 | 31.76 |  |
| Left Atrium maximum volume (mL) | Control | 50 | 35.00 | 110.80 | 72.09 | 2.06 | 70.81 | 70.81 | 77.20 | -1.68** (0.094) |
|  | Diabetic | 47 | 43.30 | 125.00 | 69.07 | 2.11 | 63.40 | 70.81 | 70.81 |  |

Min, minimum; Max, maximum; SEM, standard error of the mean; P25, percentile 25; P75, percentile 75; STS, Standardized Test Statistic obtained for the independent samples t- test (*) or for the Mann-Whitney U test (**)
