# Supplementary material for: Relations between Cardiac and Visual Phenotypes in Diabetes: A Multivariate Approach
Source: PLoS One. 2016 Apr 18;11(4):e0153772. doi: 10.1371/journal.pone.0153772 (PMC4835099; doi:10.1371/journal.pone.0153772)
Supplement: S4 Table — (DOCX) [file pone.0153772.s004.docx]

**S4 Table. DA and LR Model coefficients**

|  | VS | | RNFL | | SPEED | | ACHROM | | CHROM-P | | CHROM-D | | CHROM-T | | True-FISP | |
| --- | --- | --- | --- | --- | --- | --- | --- | --- | --- | --- | --- | --- | --- | --- | --- | --- |
|  | DS_1_ | LR_1_ | DS_2_ | LR_2_ | DS_3_ | LR_3_ | DS_4_ | LR_4_ | DS_5_ | LR_5_ | DS_6_ | LR_6_ | DS_7_ | LR_7_ | DS_8_ | LR_8_ |
| Centroid C | -0.802 | - | -0.825 | - | -0.834 | - | -0.790 | - | -0.767 | - | -0.775 | - | -0.846 | - | -0.900 | - |
| Centroid D | 0.853 | - | 0.877 | - | 0.887 | - | 0.841 | - | 0.816 | - | 0.825 | - | 0.900 | - | 0.958 | - |
| b_0_ | -9.012 | -12.177 | -8.277 | -12.731 | -7.063 | -13.956 | -7.312 | -11.984 | -7.144 | -11.003 | -6.524 | -9.885 | -6.652 | -10.631 | -4.028 | -4.290 |
| b_1j_ | 1.303 | 1.511 | 1.466 | 2.030 | 1.237 | 1.657 | 1.680 | 2.198 | 1.441 | 1.870 | 1.552 | 1.965 | 1.472 | 1.847 | 1.431 | 2.346 |
| b_2j_ | 0.254 | 0.583 | -0.009 | 0.020 | 0.063 | 0.159 | 0.018 | 0.176 | 0.020 | 0.139 | -0.104 | -0.233 | -0.161 | -0.336 | 0.410 | 1.072 |
| b_3j_ | 0.058 | 0.096 | 0.054 | 0.086 | 0.038 | 0.080 | 0.030 | 0.050 | 0.039 | 0.044 | 0.033 | 0.042 | 0.027 | 0.040 | 0.026 | 0.152 |
| b_4j_ | 0.155 | 0.251 | 0.137 | 0.221 | 0.154 | 0.280 | 0.135 | 0.230 | 0.156 | 0.254 | 0.139 | 0.218 | 0.138 | 0.230 | 0.153 | 1.593 |
| b_5j_ | -0.001 | -0.002 | 0.059 | 0.220 | 0.341 | 0.868 | -0.161 | -0.180 | 116.946 | 378.585 | 35.364 | 182.485 | 4.091 | 13.157 | -0.039 | -0.090 |
| b_6j_ | -0.009 | -0.023 | 0.007 | -0.026 | -0.238 | -0.141 | 0.177 | 0.264 | 5.624 | 3.943 | -5.909 | -13.395 | 0.402 | -1.856 | 0.042 | 0.043 |
| b_7j_ | 0.019 | 0.070 | -0.015 | -0.040 | -0.274 | -0.604 | 0.315 | 0.488 | 38.699 | 45.691 | 9.779 | 7.331 | 0.530 | 2.931 | -0.014 | -0.030 |
| b_8j_ | -0.023 | -0.058 | -0.007 | -0.029 | 0.274 | 0.606 | 0.142 | 0.171 | -67.098 | -92.866 | 8.883 | 17.766 | 4.427 | 6.124 | -0.001 | < 0.001 |
| b_9j_ | -0.012 | -0.036 | 0.022 | 0.005 | - | - | - | - | - | - | - | - | - | - | -0.024 | -0.049 |
| b_10j_ | 0.008 | 0.034 | -0.013 | -0.036 | - | - | - | - | - | - | - | - | - | - | 0.064 | 0.180 |
| b_11j_ | 0.016 | 0.001 | -0.020 | -0.047 | - | - | - | - | - | - | - | - | - | - | -0.004 | 0.001 |
| b_12j_ | 0.003 | 0.020 | - | - | - | - | - | - | - | - | - | - | - | - | 0.009 | 0.098 |
| b_13j_ | 0.005 | -0.006 | - | - | - | - | - | - | - | - | - | - | - | - | 0.009 | -0.006 |

Centroid C, centroid for group control obtained by discriminant analysis; Centroid D, centroid for type 2 diabetic group, obtained by discriminant analysis
